# Letter to the editor: spontaneous renal haemorrhage in end-stage renal disease

**DOI:** 10.1007/s13244-015-0439-4

**Published:** 2015-10-15

**Authors:** Massimo Tonolini, Anna Maria Ierardi, Gianpaolo Carrafiello

**Affiliations:** Department of Radiology, “Luigi Sacco” University Hospital, Via G.B. Grassi 74, 20157 Milan, Italy; Interventional Radiology, Department of Radiology, University of Insubria, Viale Borri 57, 21100 Varese, Italy

**Keywords:** End-stage renal disease, Haemodialysis, Haemorrhage, Computed tomography (CT), Transarterial embolisation

Dear Sir,

We read with interest the comprehensive review of renal and extrarenal findings in haemodialysis patients by Degrassi et al., recently published in *Insights into Imaging* [1]. In this paper, the authors comprehensively describe acquired cystic kidney disease (ACKD) and renal tumours in end-stage renal disease (ESRD) plus musculoskeletal, cardiovascular and miscellaneous extrarenal complications of haemodialysis. After mentioning the common haemorrhagic diathesis in patients with chronic renal failure, the authors state that “spontaneous non-traumatic bleeding may affect the perinephric and subcapsular spaces, renal parenchyma or collecting system” [1].

Upon finishing reading the article, we thought that emphasizing the issue of spontaneous bleeding in ESRD could be useful for most general radiologists. In patients on chronic haemodialysis, acute abdominal pain is a common presentation which may herald a medical or surgical emergency and carries a high risk of morbidity and mortality. Knowledge of the aetiologic spectrum of abdominal complaints and prompt imaging investigation are crucial to providing a correct and timely diagnosis: according to a recent retrospective study, in the haemodialysis population, the main causes of acute abdomen pain are spontaneous intra-abdominal haemorrhage (21.2 % of patients) and non-occlusive mesenteric ischemia (18.1 %) in descending order of frequency. Non-traumatic bleeding involving the intra-abdominal organs, retroperitoneum or muscles is significantly more common in ESRD than in the general population, invariably associated with haemodialysis rather than with peritoneal dialysis (PD), and is potentially fatal. Conversely, intestinal perforation and peritonitis largely predominate in patients on PD, and the incidence of acute pancreatitis in ESRD does not significantly differ from that in the general population [2–4].

Initially described by Wunderlich in 1856, spontaneous renal haemorrhage (SRH) with blood dissecting into the subcapsular and/or perinephric spaces is an uncommon but well-known urological emergency. In the general population, the majority (two-thirds) of occurrences are secondary to ruptured benign (angiomyolipoma) or malignant kidney tumours. Another 20–30 % of cases are related to vascular lesions such as polyarteritis nodosa, renal aneurysms or arterio-venous malformations. Occasionally, SRH results from pyelonephritis, therapeutic anticoagulation or bleeding diathesis, and 5–10 % of cases are considered idiopathic [5–8].

Conversely, in patients with chronic kidney failure, SRH is not exceptional and most usually associated with ACKD. At our hospital, where approximately 130 people receive regular haemodialysis, after excluding occurrences secondary to hereditary polycystic kidney and those attributed to excessive therapeutic anticoagulation, we collected seven such cases in ESRD (five out of seven patients were on haemodialysis) over the last 8 years. The multifactorial pathogenesis of SRH involves arterial intimal fibrosis and hypertension, causing the rupture of unsupported sclerotic arteries within cyst walls. Anticoagulation used for haemodialysis and uraemia-associated functional platelet abnormalities probably act as contributing factors [9–11].

SRH may present with the classical triad of acute lumbar or abdominal pain, a palpable mass and hypovolemic shock. Alternatively, manifestations may be nonspecific with variable degrees of haemodynamic compromise. Practically, SRH should be strongly suspected in every patient with ESRD (particularly on haemodialysis) presenting with more or less severe abdominal or flank pain, hypotension and haematocrit drop, and prompt imaging investigation is imperative. Ultrasound may promptly identify abnormal perinephric collections compressing or displacing the kidney, with decreasing echogenicity during their temporal evolution. However, ultrasound has limited diagnostic accuracy because of patient-related technical factors and does not reliably differentiate echogenic clotted blood from solid tissue [5–8].

**Fig. 1:**
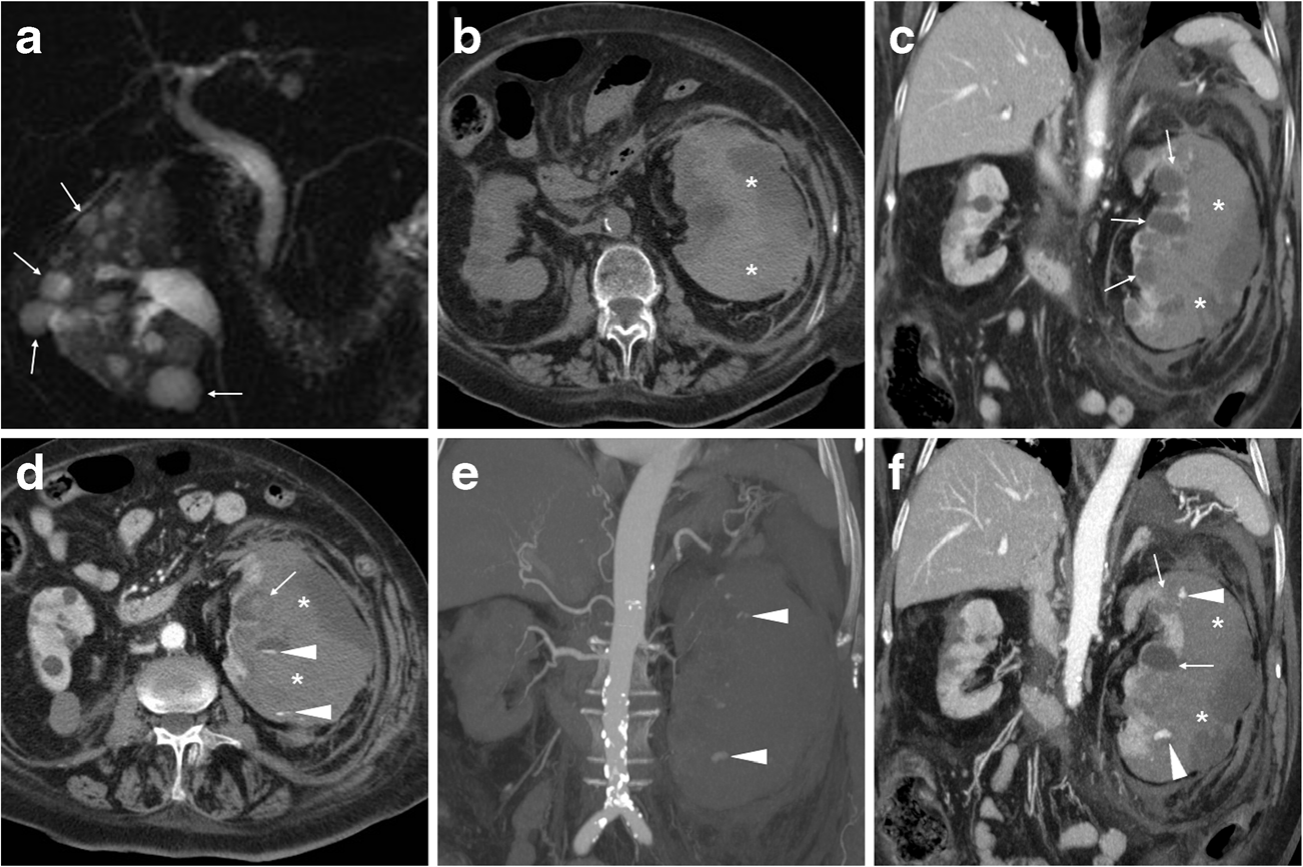
A 76-year-old female with several comorbidities, including hypertension, type II diabetes, epilepsy and chronic anaemia, suffered from severe pain and tenderness in her left lower abdomen. Features consistent with acquired cystic renal disese (ACKD) were noted in a magnetic resonance (MR)-cholangiopancreatography study (**a**) performed a few months earlier, including several moderately-sized cysts (*thin arrows*). Despite worsening end-stage renal disease (ESRD), she was not on haemodialysis and received antithrombotic prophylaxis after a previous deep venous thrombosis. Laboratory assays revealed a mild haemoglobin (8.2 g/dl) drop compared to baseline. At emergency department admission, an unenhanced multidetector CT study (**b**) was requested to investigate suspected acute diverticulitis. After detection of large left-sided subcapsular haematoma (*), a CT study was completed with contrast medium (CM) injection. Corticomedullary (**c**, **d**) and nephrographic (**f**) phase images showed the haematoma (*) exerting severe compression on the renal parenchyma, largely replaced by cysts (*thin arrows*) with mural discontinuity. Complemented with angiographic maximum-intensity projection (MIP) reconstructions (**e**), CT visualized small foci of CM extravasation isoattenuating with the blood pool (*arrowheads*). During renal arteriography (not shown), active bleeding was not observed anymore, indicating its spontaneous cessation. The patient slowly recovered during intensive care unit hospitalization, including blood transfusions and correction of metabolic acidosis

**Fig. 2:**
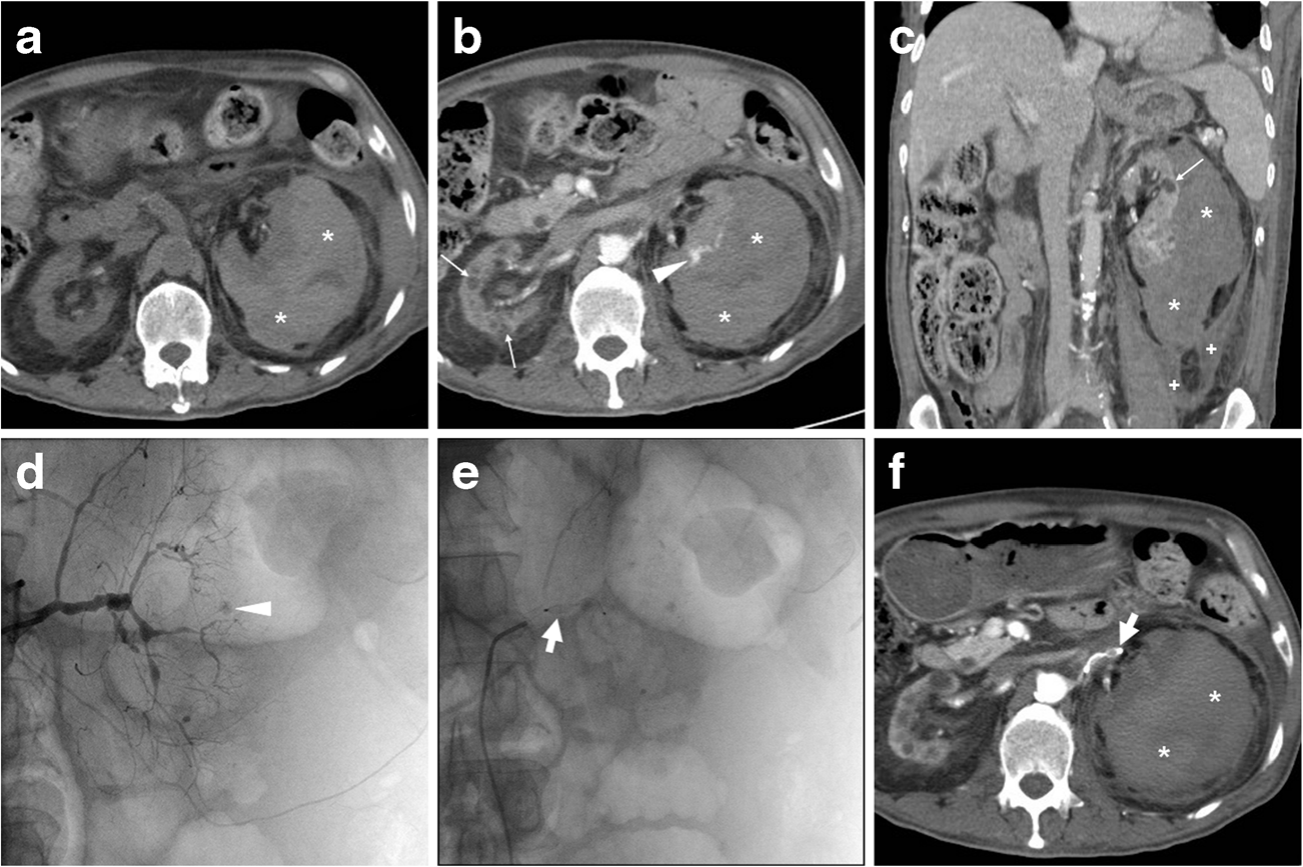
A 56-year-old Chinese man with hypertension, diabetes and chronic kidney failure undergoing regular haemodialysis suffered from acute abdomen pain with hypotension. Unenhanced (**a**), corticomedullary (**b**) and nephrographic (**c**) multidetector CT acquisitions showed a large left-sided subcapsular renal haematoma (*) and typical features of ESRD, including small cortical cysts (*thin arrows*). Note the minimal associated perirenal and posterior pararenal bloody effusion (+ in **c**). Focal CM extravasation consistent with active bleeding was detected by CT (*arrowhead* in **b**) and confirmed at selective renal angiography (*arrowhead* in **d**), originating from a distal arterial branch at the lower half of the kidney. Angiography revealed severe luminal irregularities of all renal arteries and allowed occlusion of the left renal artery with an 8-mm Amplatzer vascular plug (AGA Medical Corp., Plymouth, MN: *short arrows* in **e**) distally to the inferior adrenal artery. Follow-up CT (**f**) confirmed the Amplatzer plug in site (*short arrow*) and stopped haemorrhage

Multidetector computed tomography (CT) represents the imaging modality of choice to diagnose suspected intra-abdominal bleeding and differentiate from other causes of acute abdomen. CT has absolute (100 %) sensitivity for the detection of retroperitoneal haematomas, which appear as more or less hyperattenuating (35 to 70 Hounsfield units) collections on precontrast scans depending on their more or less acute stage. In our experience, the typical appearance of SRH in ACKD includes small, poorly perfused kidneys with multiple cysts and a sizeable crescent- or biconvex-shaped subcapsular haematoma exerting compression on the adjacent renal parenchyma (Figs. 1 and 2). CT allows accurate size measurement and monitoring of haematomas, and differentiates subcapsular from perinephric haematomas which displace the kidney ventrally without compression. Furthermore, multiphase protocols, including corticomedullary and nephrographic acquisitions, allow identification of contrast medium (CM) extravasation isodense with the blood pool consistent with active bleeding (Figs. 1 and 2) [5, 7, 8, 10, 12].

In the past, radical nephrectomy was recommended in SRH due to the increased risk of renal cell carcinoma (RCC) in haemodialysis patients, which is 3 to 13-fold higher than matched control subjects. Careful scrutiny of imaging studies for solid masses and CT or MR imaging follow-up is recommended to exclude RCC underlying SRH. The reported sensitivity of CT for the detection of renal masses or vascular abnormalities causing SRH varies between 57 % and 100 % [5, 7, 8, 10]. According to our personal experience, this concern is probably overstated because most haemodialysis patients undergo periodic sonographic surveillance, and none of our patients had underlying RCC [1].

Currently, a conservative therapeutic approach (bed rest, transfusion support and withdrawal of anticoagulation) is recommended and generally successful in haemodynamically stable patients, provided that the initial CT does not show renal masses, aneurysms and CM extravasation. In patients with clinical, laboratory and imaging signs of ongoing haemorrhage, selective renal angiography with transcatheter embolisation (Fig. 2) using various embolic materials is increasingly performed and may represent the ideal minimally invasive therapeutic approach for SRH [11–15].
